# Predicting pathological subtypes of pure ground-glass nodules using Swin Transformer deep learning model

**DOI:** 10.1186/s13244-025-02113-3

**Published:** 2025-10-17

**Authors:** Yanhua Wen, Menna Allah Mahmoud, Wensheng Wu, Huicong Chen, Yingying Zhang, Xiaohuan Pan, Yubao Guan

**Affiliations:** 1 Department of Medical Imaging, The Fifth Affiliated Hospital of Guangzhou Medical University, Guangzhou, China; 2 Guangzhou Medical University, Guangzhou, China; 3 General Hospital of the Southern Theatre of the Chinese People’s Liberation Army, Guangzhou, China; 4 Department of Radiology, The First Affiliated Hospital of Guangzhou Medical University, Guangzhou, China

**Keywords:** X-ray computed tomography, Deep learning, Ground-glass opacity, Lung adenocarcinoma, Pathological subtypes

## Abstract

**Objectives:**

To explore the diagnostic value of a multi-classification model based on deep learning in distinguishing the pathological subtypes of lung adenocarcinoma or glandular prodromal lesions with pure ground-glass nodules (pGGN) on CT.

**Materials and methods:**

A total of 590 cases of pGGN confirmed by pathology as lung adenocarcinoma or glandular prodromal lesions were collected retrospectively, of which 462 cases of pGGN were used as training and testing set, and 128 cases of pGGN as external verification set. The research is based on the Swin Transformer network and uses a five-fold cross-validation method to train the model. The diagnostic efficacy of deep learning model and radiologist on the external verification set was compared. The classification efficiency of the model is evaluated by confusion matrix, accuracy, precision and F1-score.

**Results:**

The accuracy of the training and testing sets of the deep learning model is 95.21% and 91.41% respectively, and the integration accuracy is 94.65%. The accuracy, precision and recall rate of the optimal model are 87.01%, 87.57% and 87.01% respectively, and the F1-score is 87.09%. In the external verification set, the accuracy of the model is 91.41%, and the F1-score is 91.42%. The classification efficiency of the deep learning model is better than that of radiologists.

**Conclusion:**

The multi-classification model based on deep learning has a good ability to predict the pathological subtypes of lung adenocarcinoma or glandular prodromal lesions with pGGN, and its classification efficiency is better than that of radiologists, which can improve the diagnostic accuracy of pulmonary pGGN.

**Critical relevance statement:**

Swin Transformer deep learning models can noninvasively predict the pathological subtypes of pGGN, which can be used as a preoperative auxiliary diagnostic tool to improve the diagnostic accuracy of pGGN, thereby optimizing the prognosis of patients.

**Key Points:**

- The Swin Transformer model can predict the pathological subtype of pure ground-glass nodules.
- Compared with the performance of radiologists, the deep learning model performs better.
- Swin Transformer model can be used as a tool for preoperative diagnosis.

**Graphical Abstract:**

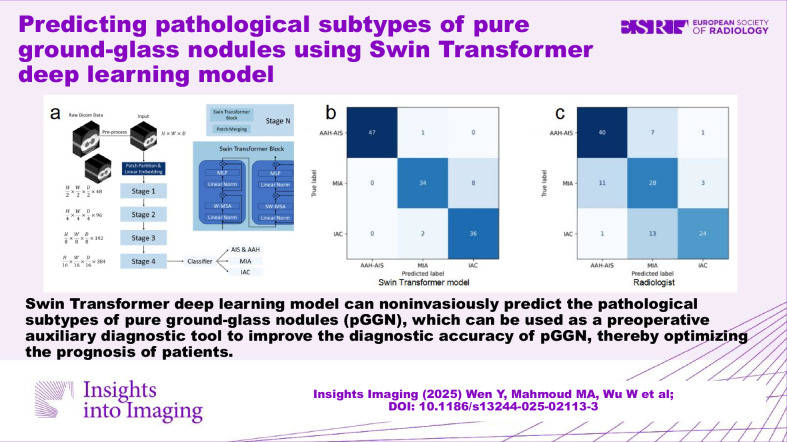

## Introduction

Lung cancer is one of the most common and serious causes of cancer-related deaths worldwide [1]. Lung adenocarcinoma is the most common histological type of lung cancer [2]. In the new World Health Organization (WHO) classification of lung adenocarcinoma in 2021, lung epithelial tumors are classified as glandular precursor lesions (GPLs), minimally invasive adenocarcinoma (MIA), and invasive adenocarcinoma (IAC), of which GPLs, which include atypical adenomatous hyperplasia (AAH) and adenocarcinoma in situ (AIS), are recommended for regular observation in clinical practice, while MIA and AIC require surgical intervention [2, 3].

Pure ground-glass nodules (pGGNs) can be defined as nodules with a hazy attenuation increase in the lung window, which do not obscure the blood vessels and bronchial structures, and cannot be visualized in the mediastinal window [4]. With the improvement of people’s awareness of health check-ups, the detection rate of pGGN has significantly increased [5]. Persistent pGGNs (> 3 months) are closely related to early lung adenocarcinoma or precancerous lesions, and generally follow the progression from AAH to AIS to MIA and finally to AIS [5–7]. Most pGGN are pre-invasive lesions; however, recent histological studies have shown that persistent pGGN have an infiltrative component and that 30–40% of patients with resected pGGN are IAC [7, 8]. Therefore, there is an overlap in the imaging features of the different histopathological subtypes that manifest as pGGN on CT.

Follow-up observation can be considered for pGGN with suspected AAH and AIS, because although the 5-year survival rate after operation is 100%, the lung function of these patients has decreased [9, 10]. For pGGN with a pathological diagnosis of MIA, sublobectomy (wedge or segmental lobectomy) can be performed, and the 5-year survival rate can be close to 100% [10]. Lobectomy is still considered the standard surgical treatment for IAC. Due to the invasive biological behavior of IAC tumor cells, the 5-year disease-free survival rate (40–85%) and quality of life decreased significantly [9–11]. Therefore, accurate preoperative assessment of the invasiveness of pGGN, especially for young patients or those with high-risk factors (such as a family history of lung cancer), has important clinical significance in the selection of treatment regimens and the optimization of patient prognosis. However, at present, the diagnosis of pGGN mainly relies on the visual assessment of radiologists, which is easily affected by subjective factors and diagnostic experience, and its diagnostic accuracy needs to be improved [12–14].

Through autonomous learning and self-optimization, deep learning can speed up the process of data analysis and feature extraction, build a prediction model with stronger generalization ability, and the research results are relatively less disturbed by subjective factors [15–17]. Transformer is a deep learning model based on a self-attention mechanism, which has the advantage of processing long sequential data and enabling parallelized computation compared to neural network models [18]. Swin Transformer is a novel Transformer architecture designed for computer vision tasks, which introduces a moving-window-based self-attention mechanism and employs a hierarchical feature representation, allowing the model to strike a balance between computational complexity and performance [19].

To the best of our knowledge, there are no previous studies applying Swin Transformer-based deep learning techniques to evaluate the pathological histological subtypes of pGGN. Therefore, in order to preoperatively and accurately assess AAH & AIS, MIA, and IAC that manifested as pGGN on CT, this study constructs a deep learning triple classification model based on the Swin Transformer architecture and externally validates it with data from different healthcare institutions to further optimize the model’s robustness and generalization, with the aim of using it as a preoperative auxiliary diagnostic tool to improve the pGGN diagnostic accuracy, thereby optimizing patient prognosis.

## Materials and methods

Our trial protocol conforms to the ethical guidelines of the Declaration of Helsinki and was approved by the Ethics Review Committees of three institutions, the approval numbers are KY01-2022-02-14, 2024-195KY, and (MER) 2017-38. Because of the retrospective nature of the study, the need for informed consent was waived by the institutional review board.

### Study population

**Fig. 1:**
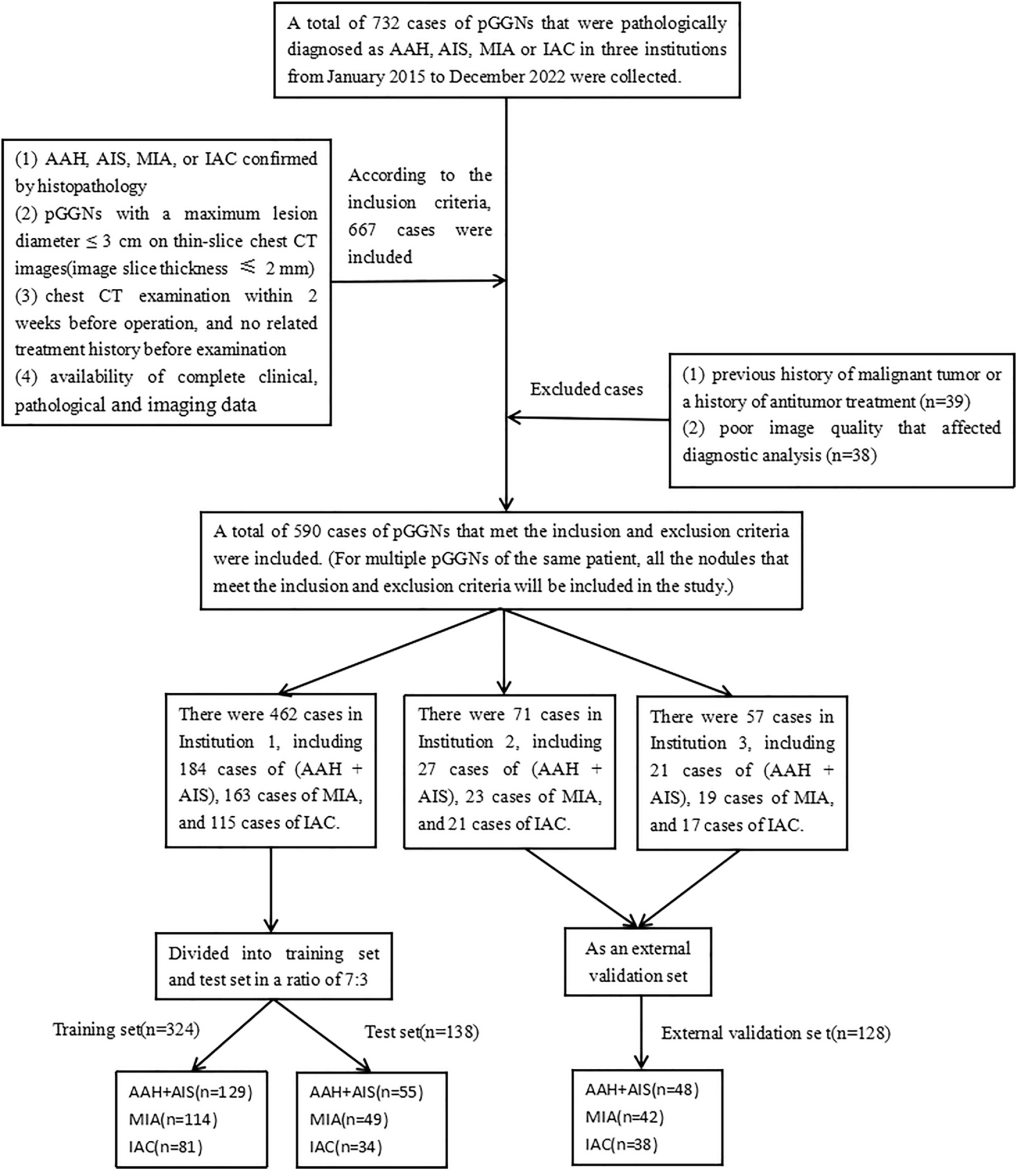
The screening process of the subjects

The clinical, pathological, and imaging data of 590 patients with pulmonary pGGNs confirmed as AAH, AIS, MIA, or IAC by surgical resection and pathological examinations from three hospitals from January 2015 to December 2022 were collected retrospectively. The criteria for collecting pGGNs are as follows: nodules with increased attenuation are presented in the lung window, but these nodules do not obstruct the vascular and bronchial structures, and they cannot be clearly displayed in the mediastinal window [20]. The inclusion criteria were as follows: (1) AAH, AIS, MIA, or IAC confirmed by surgical resection and histopathology; (2) pGGNs with a maximum lesion diameter ≤ 3 cm on thin-slice chest CT images (image slice thickness ≤ 2 mm); (3) chest CT examination within 2 weeks before operation, and no related treatment history before examination; (4) availability of complete clinical, pathological and imaging data. The exclusion criteria were as follows: (1) previous history of malignant tumor or a history of antitumor treatment; (2) poor image quality that affected diagnostic analysis. Figure 1 shows the screening process of the subjects in this study.

According to the inclusion and exclusion criteria, a total of 590 pGGNs were included in this study, including 462 cases from institution 1 for the training and internal validation of the model, and 128 cases from institution 2 and institution 3 for the external validation set.

### CT scanning scheme

Images were acquired at the three hospitals with Siemens Definition AS + 128-slice CT, Canon 640-slice CT (Aquilion ONE Vision), and GE Optima CT660 128-slice CT scanners. The scanning parameters were as follows: tube voltage, 120 kV; automatic millisecond current; field of view, 400 × 400 mm; matrix, 512 × 512; thickness, 1.0 mm; and pitch, 1.0. Images were reconstructed using a 1.0-mm layer thickness standard algorithm and lung algorithm. All CT images were exported in DICOM format for backup.

### Pathological diagnostic criteria

**Fig. 2:**
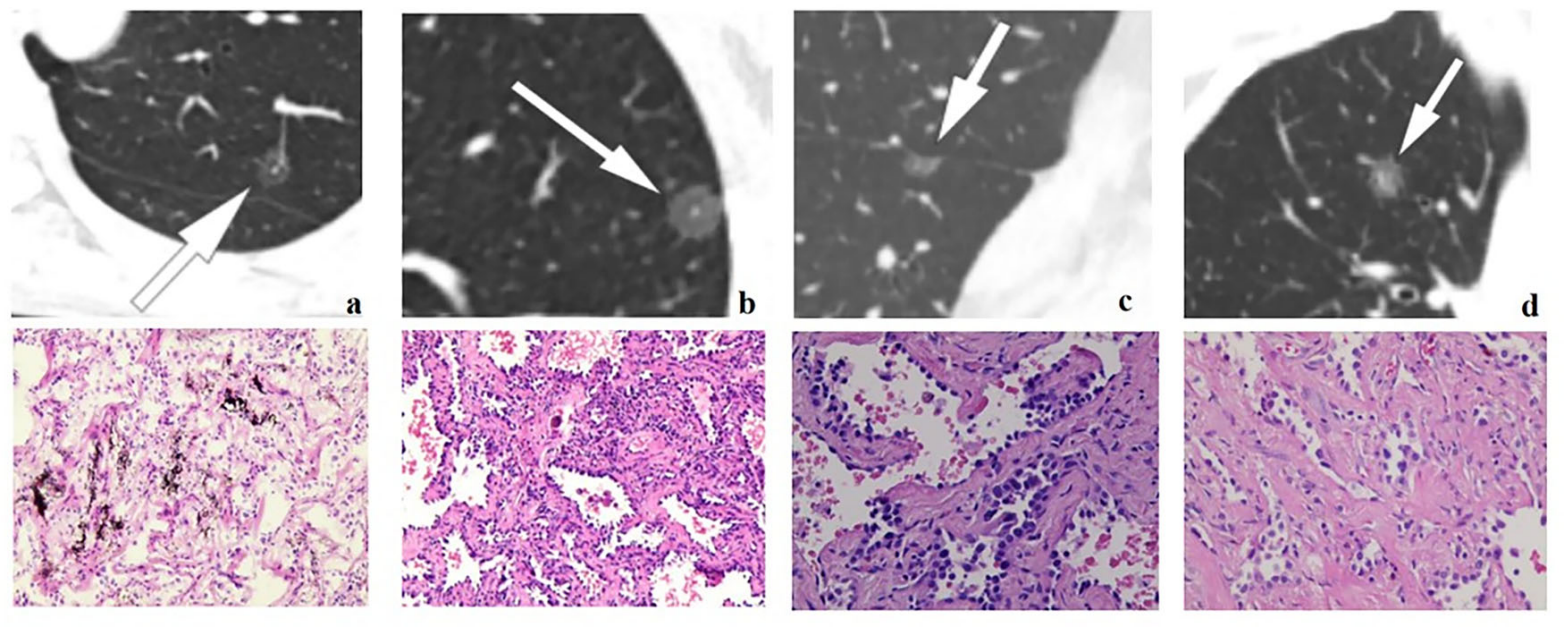
The CT images of AAH, AIS, MIA and IAC and their corresponding pathological sections. **a**–**d** The CT images of AAH, AIS, MIA, and IAC, respectively, and the corresponding lower images show the pathological images. As can be seen from the above figure, pure ground-glass nodules that appear similar on CT images have different pathological findings (the high-density area within the nodule in the above figure was confirmed to be vascular track signs rather than a solid component on serial tomography images), which is difficult to identify by visual assessment by the radiologist alone

All pGGN specimens were obtained after surgical resection. After fixation, dehydration, entrapment, sectioning, hematoxylin-eosin staining, and sealing, experienced pathologists observed the specimens under light microscopy with different magnification levels. The diagnostic criteria were based on the 2021 WHO lung cancer classification guidelines [2]. In accordance with the 2021 classification standard of lung adenocarcinoma, AIS was removed from the subdirectory of lung adenocarcinoma, while AAH and AIS were classified as glandular precursors. Figure 2 shows CT images of AAH, AIS, MIA, and IAC and their corresponding pathological sections.

### Experimental conditions and implementation

Hardware: Intel® Xeon® Gold 5218 CPU@2.30GHz*2, 128GiB System memory, Nvidia RTX TITAN*3. Software: Ubuntu 20.04, Python 3.9.13, MONAI 1.1.0, Torch 1.12.1, Scikit-learn 1.0.2, Imple ITK 2.2.1, Matplotlib 3.5.2.

In the data preprocessing, the two-dimensional time series DICOM format files are converted into three-dimensional images in Nifti format, and the voxel values are normalized according to the window width (1400 HU) and window level (−500 HU) used by doctors to observe lung tumors. Due to the use of multi-center data, we rearranged the space of each image to set the distance of voxels uniformly. Because the amount of data is not large enough, the data enhancement methods of random flipping and random rotation are adopted in this study.

The model is based on the Swin Transformer structure, which is composed of patch embedding layer and four stages. The input 3D images are divided into non-overlapping token in the patch embedding layer, and sequence is formed according to the original relationship between them.

Each stage contains a complete Swin Transformer block and a patch merging layer. The Swin Transformer block is composed of a window multi-head self-attention module and a sliding window self-attention module, which are connected in series. Using the formula, 3D Swin Transformer block can be expressed as:

$${\hat{z}}^{l}=W-{MSA}({LN}({z}^{l-1}))+{z}^{l-1}$$

$${z}^{l}={MLP}({LN}({\hat{z}}^{l}))+{\hat{z}}^{l}$$

$${\hat{z}}^{l+1}={SW}-{MSA}({LN}({z}^{l}))+{z}^{l}$$

$${z}^{l+1}={MLP}({LN}({\hat{z}}^{l+1}))+{\hat{z}}^{l+1}$$

The self-attention mechanism can be expressed as:

$${Self}-{Attention}(Q,K,V)={SoftMax}\left(\frac{Q{K}^{T}}{\sqrt{d}}\right)V$$

### Experimental details

**Fig. 3:**
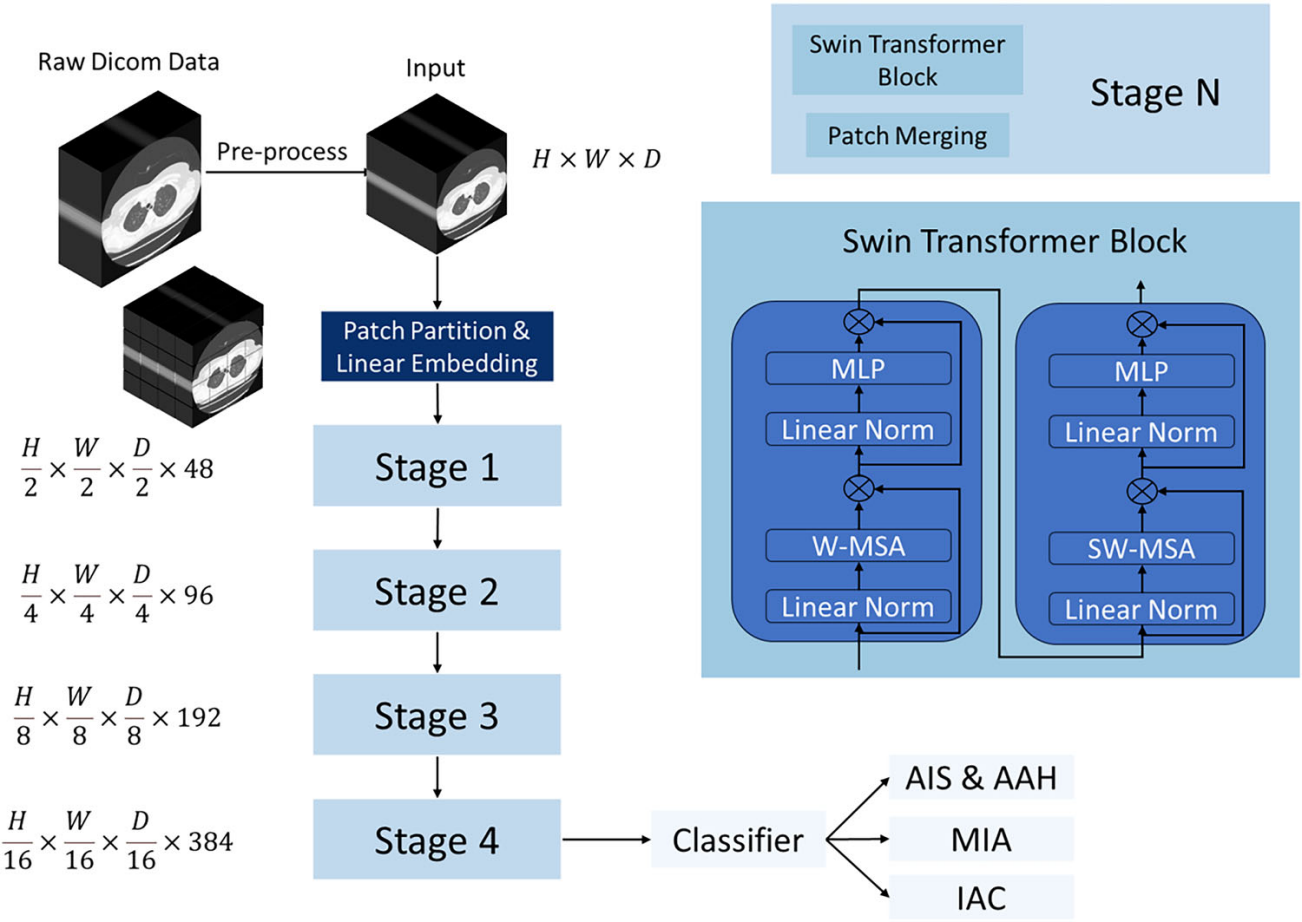
Swin Transformer model network structure

Using the parameters of self-supervised pre-training of 5050 CT images in Swin UNETR, transfer learning is carried out and loaded into our model structure. The optimizer uses AdamW to initialize the learning rate of 0.01, weight decay, and selects Exponential for 1eMurray, and the learning rate of each epoch attenuates to 0.95. Because of the quantitative difference between the data, the loss function adopts weighted BCE loss. The batch size is set to 4 (128 × 128 × 128 Voxel) for each video card, and the 100 epoch is trained altogether. The dataset is cross-validated according to five-folds, and the model with the highest accuracy per discount is selected, and their output is integrated into the final classification result. The network structure of the Swin Transformer model is shown in Fig. 3.

### The radiologist’s diagnosis

To compare the model performance with the radiologist’s diagnosis, the radiologists were asked to evaluate the data from an external validation set. Two radiologists with 5–10 years of experience in chest imaging diagnosis independently predicted the pathological subtype of pGGN (diagnosing it as AAH & AIS or MIA or IAC) without knowing the pathological results. When the diagnosis results are inconsistent, the two radiologists will jointly deliberate and reach a consensus on the final result.

### Statistical analysis

SPSS 24.0 (IBM) software was used for statistical analysis. The measurement data were expressed as mean ± standard deviation, and the counting data were expressed by frequency. T-tests of two independent samples were performed for comparison of measurement data, and the chi-square test was used for comparison of counting data. When *p* < 0.05, the difference was considered to be statistically significant.

Confusion matrix, accuracy, precision, weighted recall and weighted F1-score are used to evaluate the classification performance. The relevant formulas are expressed as shown in formulas (1)–(4):

1 $$Accuracy=\frac{TP+TN}{TP+FP+TN+FN}$$

2 $$Precision=\frac{TP}{TP+FP}$$

3 $$Recall=\frac{TP}{TP+FN}$$

4 $$F1-{{score}}=\frac{2\times {{Precision}}\times {{Recall}}}{{{Precision}}+{{Recall}}}$$

(TP: True positive, TN: True negative, FP: False positive, FN: False negative)

The confusion matrix uses the prediction label as the horizontal axis and the real label as the vertical axis to form the matrix diagram.

## Results

### Clinical data of patients

A total of 590 pGGN cases were included in this study, of which 462 cases were used for the training and internal validation of the model (data from institution 1), and 128 cases were used as external validation (data from institution 2 and institution 3). The general clinical data of the included patients were analyzed, including patient age and gender. The analysis results are shown in Tables 1 and 2, respectively.

**Table 1:** Clinical characteristics of the training and testing set

Clinical	Training and testing set (*n* = 462)			*F* or *χ2*	*p*-value
	AAH/AIS	MIA	IAC		
Age (years) (mean ± SD)	52.69 ± 10.97	51.42 ± 11.71	57.55 ± 11.70	7.185	0.001
Gender (male/female)	69/115	68/95	44/71	2.001	0.368

*SD* standard deviation, *AAH* atypical adenomatous hyperplasia, *AIS* adenocarcinoma in situ, *MIA* minimally invasive adenocarcinoma, *IAC* invasive adenocarcinoma

**Table 2:** Clinical characteristics of the external verification set

Clinical	External verification group (*n* = 128)			*F* or *χ2*	*p*-value
	AAH/AIS	MIA	IAC		
Age (years) (mean ± SD)	48.49 ± 10.81	49.58 ± 10.36	52.87 ± 11.08	1.549	0.217
Gender (male/female)	16/32	17/25	12/26	3.188	0.203

*SD* standard deviation, *AAH* atypical adenomatous hyperplasia, *AIS* adenocarcinoma in situ, *MIA* minimally invasive adenocarcinoma, *IAC* invasive adenocarcinoma

### Model results

**Fig. 4:**
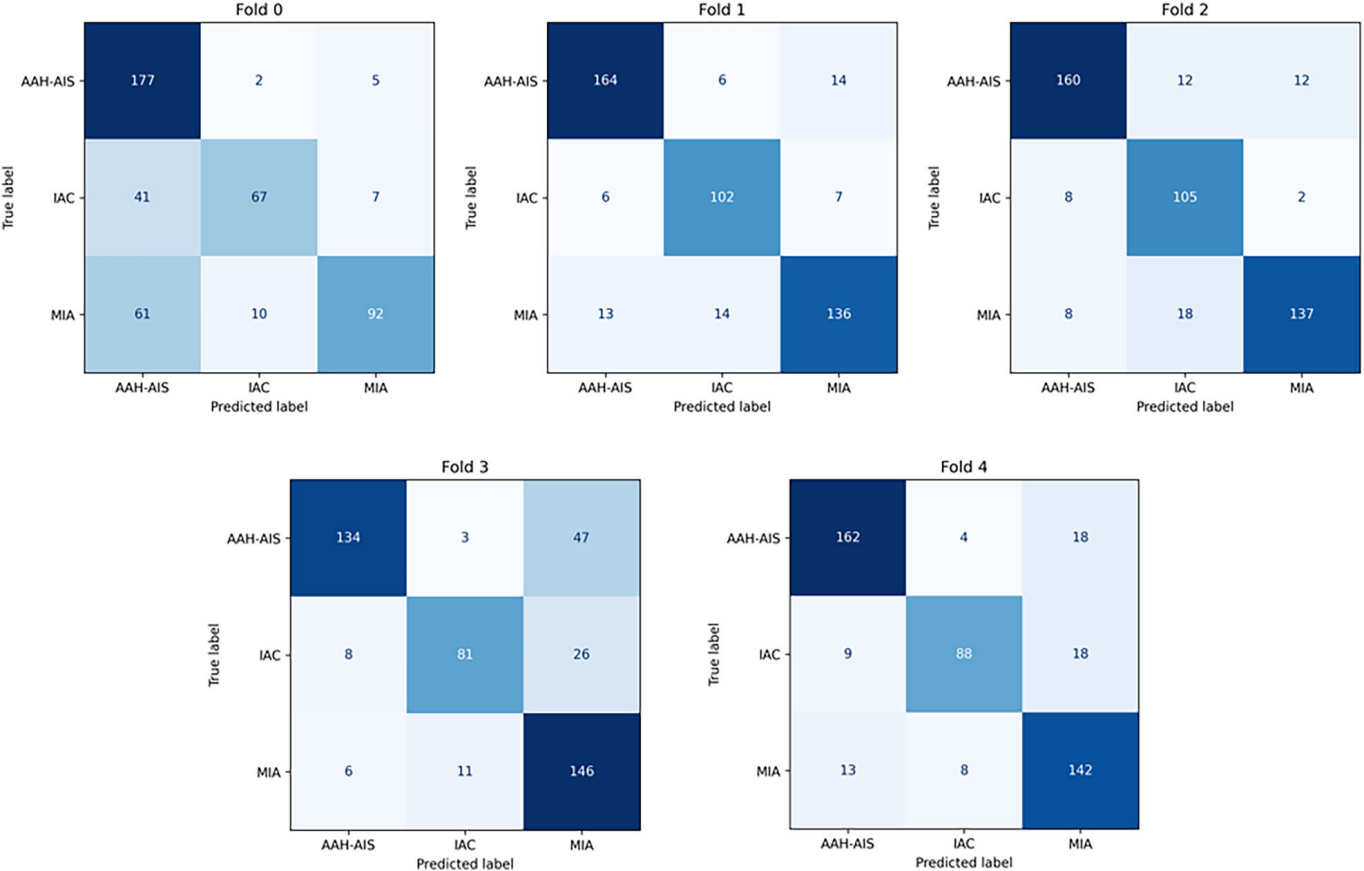
Confusion matrix results of 5-fold cross-validation of deep learning models based on Swin Transformer architecture

In our study, the deep learning model based on Swin Transformer architecture achieved good performance in identifying AAH & AIS, MIA and IAC (CT manifested as pGGN). As shown in Table 3, among the five models obtained by five-fold cross-validation, the accuracy of the optimal model is 0.8701, the precision is 0.8757, the weighted recall rate is 0.8701, and the weighted F1-score is 0.8709. The confusion matrix of the five models is shown in Fig. 4.

**Table 3:** Results of 5-fold cross-validation of deep learning models based on Swin Transformer architecture

Category	Accuracy	Precision	Recall	F1-score
Fold1	0.7273	0.7758	0.7272	0.7195
Fold2	0.8701	0.8706	0.8701	0.8700
Fold3	0.8701	0.8757	0.8701	0.8709
Fold4	0.7814	0.8080	0.7813	0.7832
Fold5	0.8485	0.8511	0.8484	0.8482

### Comparison of the results of deep learning model and radiologists

**Fig. 5:**
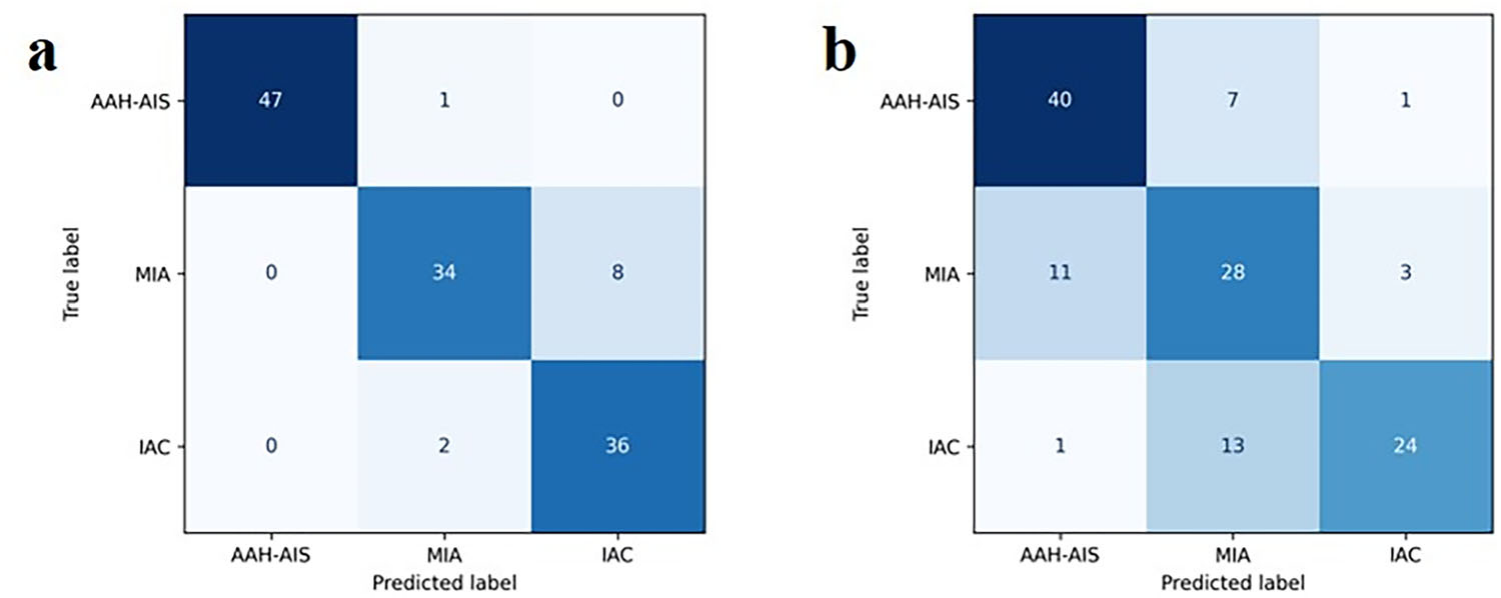
Confusion matrix results of pGGNs predictions by deep learning models and radiologists in the external validation set. In the picture, **a** and **b** respectively represent the prediction results of the deep learning model and the radiologist

The predictive performance of the model and radiologists for pGGN in the external validation set is shown in Table 4. Table 4 shows that radiologists have a certain ability to identify pathological subtypes of pGGNs, but the prediction performance of the Swin Transformer model is significantly better than that of radiologists. In addition, the accuracy, precision, Recall and F1-score of the deep learning model achieved good results, which were 0.9141, 0.9194, 0.9141 and 0.9142, respectively. The confusion matrix results of pGGNs predictions from the deep learning model and radiologists in the external validation set are shown in Fig. 5.

**Table 4:** Evaluation indicators of the external verification set

Category	Accuracy	Precision	Recall	F1-score
Model	0.9141	0.9194	0.9141	0.9142
Radiologist	0.7188	0.7343	0.7188	0.7201

### Model explainability

**Fig. 6:**
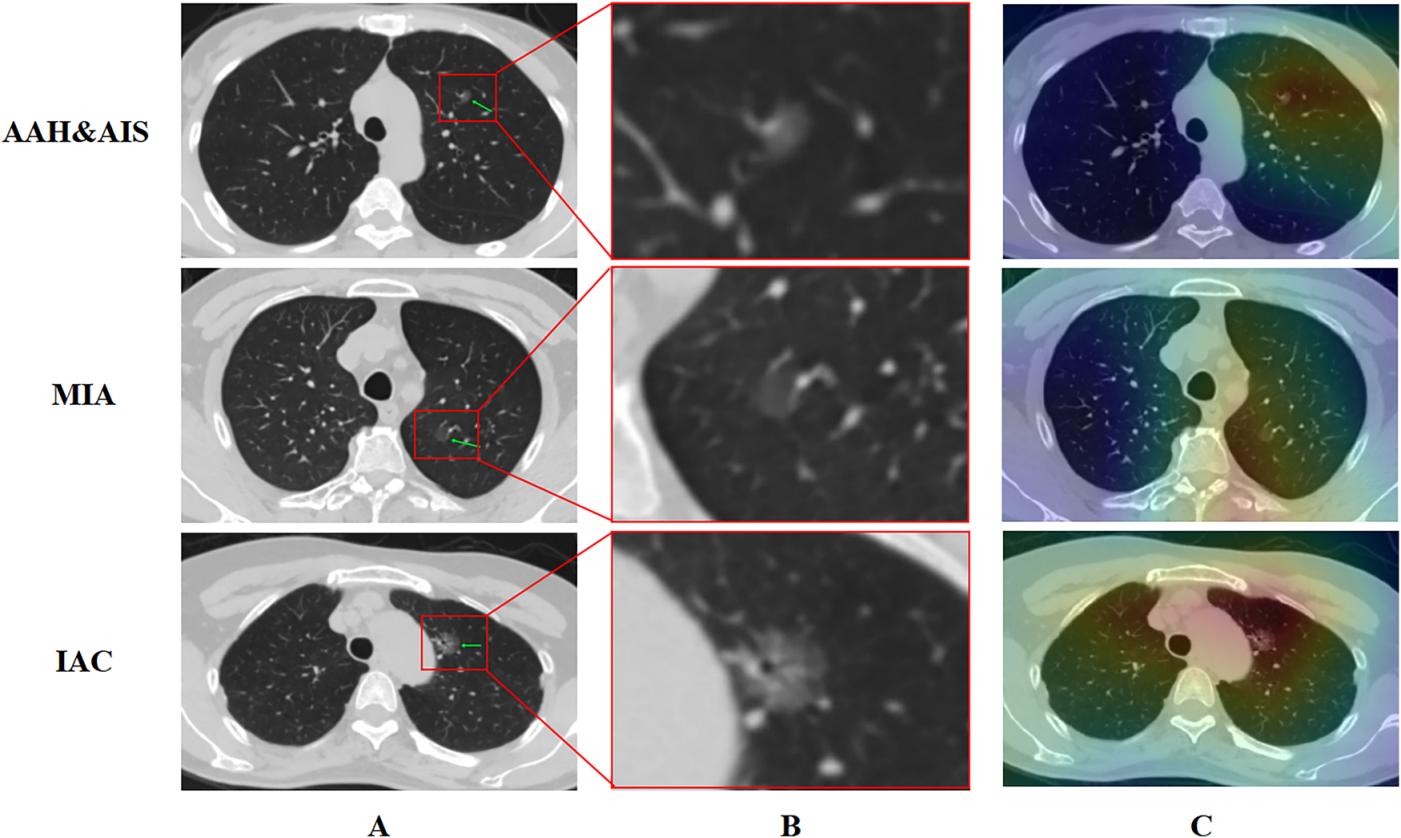
Using CAM visualization to verify model interpretability. **A** This column shows the original images, with the green arrows indicating the lesion sites for easy identification. **B** This column presents the enlarged images of the lesions (the high-density area within the nodule in the above figure was confirmed to be vascular track signs rather than a solid component on serial tomography images). **C** This column shows the visualization images, which indicate that in the three groups of AAH & AIS, MIA and IAC, the model can identify the regions where the lesions are located

To determine the lesion regions recognized by the model and to enhance its acceptance in clinical practice. We adopt the method of Class Activation Mapping (CAM) to locate the key regions in the image by using gradient information and generate visualized images. Figure 6 shows that this model can identify the regions where the lesions are located.

### Independent dataset validation and model comparison

The effectiveness of our method was further verified using MedMNIST [21] and Fracremist3d [22] (a 3D chest CT medical image dataset for multi-classification tasks). The training set and validation set consisted of 1027 and 103 cases, respectively, while the test set contained 240 cases. In the experiments using the FractureMNIST3D dataset, we also compared the benchmark performance of the benchmark (Resnet50) model and the basic Swin Transformer model with our Swin Transformer model. The parameters were kept consistent with the previous experiments, and no optimization was performed on the independent datasets. The results are shown in Table 5. The results indicate that this transformer structure outperforms the traditional convolutional neural network (Resnet50) and the basic Swin Transformer model.

**Table 5:** Comparison of results on the FractureMNIST3D dataset

	AUC	ACC
Benchmark (Resnet50)	0.725	0.494
Basic Swin Transformer	0.814	0.588
Ours	0.989	0.942

## Discussion

Although most persistent partial solid nodules are minimally invasive or invasive adenocarcinomas and show relatively obvious invasiveness, invasive adenocarcinomas can also present as pure ground-glass nodules [23, 24], which are often misdiagnosed and receive unnecessary surgery or delayed treatment due to the overlapping signs on CT and the lack of characteristic imaging manifestations. Studies showed that for lung adenocarcinoma whose preoperative CT is pGGN and intraoperative freezing is mainly adherent type, lymph node sampling rather than lymph node dissection should be considered [25]. Thus, it is of great clinical importance to make an accurate preoperative diagnosis of the pathological subtypes of pGGN. Therefore, we constructed a deep learning model based on the Swin Transformer architecture in an attempt to differentiate AAH & AIS, MIA, and IAC that manifest as pGGN on CT.

Artificial intelligence-assisted image diagnosis is based on the pixel-level data of pGGN lesions invisible to the naked eye, yielding a detailed data representation and an efficient prediction model that is less affected by human factors [26–28]. The deep learning model of this study is based on the Swin Transformer network, which is a Transformer-based backbone structure suitable for CV field put forward by Microsoft Research Asia in 2021 [29]. As one of the main representatives of the visual conversion network, this network uses Shift Window Multi-Head Self-Attention (SW-MSA) to integrate information between different panes. The advantage of the Swin Transformer network is that it is not limited by the image input size, the computational complexity is relatively low, and the classification performance of natural images is better than that of Vit network, so it is widely used in various image classification and recognition fields.

The results of this study indicate no statistically significant difference in the sex distribution among the groups with AAH & AIS, MIA, and IAC, consistent with previously published results [7]. Our results also showed statistically significant differences in age among these three groups in the training group, but no statistically significant intergroup differences in age in the external validation group. This may be related to the retrospective nature of this study and the potential for selection bias.

Previous researchers mainly predicted the aggressiveness of pGGNs based on CT signs and radiomics, and most of them were dichotomous studies, without further refinement of the data for MIA and IAC [12, 30, 31]. Previous studies on the invasiveness of pGGNs based on CT signs yielded different results. Hsu et al [12]showed that 7 mm was the best cut-off value for the average diameter of pGGN, and the sensitivity and specificity in distinguishing invasive adenocarcinoma from non-invasive adenocarcinoma were 50.0% and 76.4%, respectively. However, Qi et al [30] reported that the cut-off value of 11.5 mm for the diagnosis of pGGNs < 30 mm had a sensitivity of 75% and specificity of 91.8%. Radiologists mainly diagnose lesions according to their CT morphological characteristics (such as lobulation sign, spiculation sign, and size), and the results of such evaluations are easily affected by subjective factors, resulting in low diagnostic accuracy. Fan et al [9] showed that imaging features have good predictive ability to distinguish between invasive adenocarcinomas and non-invasive lesions. Zhao et al [31] used a three-dimensional convolutional neural network and deep learning method of multi-task learning, constructed a three-classification prediction model to identify the invasiveness of subcentimeter lung adenocarcinoma. However, none of the above-mentioned researchers were able to make a clear diagnosis of the pathological subtypes of lung adenocarcinoma or glandular prodromal lesions with pGGN. In this study, the multi-classification prediction model based on the Swin Transformer network is used to classify the pathological subtypes of pGGN on CT, and a better prediction efficiency is obtained. Through the external verification and comparison of the diagnostic results of the model and radiologists, it shows that the classification efficiency of the model is better than that of the radiologists, which shows that the deep learning model can be used as an auxiliary tool to improve the accurate diagnosis of pGGN pathological subtypes by radiologists and provide guidance for clinical diagnosis and treatment.

Although the Swin Transformer deep learning model performed well in this study, integrating it into clinical workflows still faces many challenges. (1) First, how to integrate the model with the radiological workflow? The radiological workflow is complex, and the model needs to be seamlessly connected with existing imaging equipment, PACS systems, etc. Moreover, the integration may change the routine working mode of radiologists, such as adding model invocation and result interpretation steps, which may lead to workflow chaos. During actual operation, specialized software interfaces or plugins can be developed to enable deep integration of the model with the PACS system, achieving automatic image transmission, automatic model invocation, and automatic result return. At the same time, training radiologists to familiarize themselves with the usage process and result interpretation of the model can reduce interference with the workflow. (2) Second, how to address the limitations brought by the variability of multi-center data, as different centers have differences in imaging equipment, scanning parameters, image quality, etc., resulting in large variability in data and affecting the generalization ability of the model. In addition, the diagnostic criteria and clinical practices of each center may also be different, further increasing the complexity of model application. To solve this problem, we believe that establishing a multi-center data-sharing platform, collecting and organizing standardized imaging data for model training and validation, is necessary. At the same time, adopting data augmentation techniques and transfer learning methods can improve the model’s adaptability to different data distributions. Moreover, for the special needs of different centers, the model can be fine-tuned or customized. (3) Finally, the use of the model faces issues of cost-effectiveness and diagnostic efficiency: the development and deployment of the model require certain hardware and software investments, including high-performance computing equipment, data storage facilities, and software development and maintenance costs. However, in the long run, it can reduce unnecessary biopsies and surgeries and lower medical costs. Moreover, the use of the model can improve diagnostic accuracy, reduce misdiagnosis, and enhance medical quality and patient satisfaction. Additionally, the model can analyze and diagnose a large amount of imaging data in a short time, significantly improving diagnostic efficiency.

Several limitations should be noted. (1) First, this study has selection bias. On the one hand, it is a retrospective study. On the other hand, the data is concentrated in a single country, which may lead to an overrepresentation of certain diseases or a population-based bias. This bias can be mitigated by integrating diverse data from different regions or more hospitals to ensure the diversity and representativeness of the sample. (2) Second, pGGN can also be focal inflammation, alveolar hemorrhage or fibrosis in addition to GPL, MIA and IAC. Subsequent studies can include more types of pGGN to improve the generalization of the model. (3) Finally, in this study, we only analyzed the clinical characteristics of age and gender. In future studies, we will accumulate more cases and supplement more clinical features to enhance the practicality of these clinical characteristics.

## Conclusion

The deep learning model showed good diagnostic efficacy for pathological subtyping of pure ground-glass nodules, and the diagnostic efficacy of the model was better than radiologists. This model can be used for non-invasive and individual evaluation of lung adenocarcinoma and glandular prodromal lesions manifesting as pure ground-glass nodules.

## Data Availability

The dataset used or analyzed during the current study is available from the corresponding author upon reasonable request.

## Abbreviations

AAH: Atypical adenomatous hyperplasia
AIS: Adenocarcinoma in situ
FN: False negative
FP: False positive
IAC: Invasive adenocarcinoma
MIA: Minimally invasive adenocarcinoma
pGGN: Pure ground-glass nodule
TN: True negative
TP: True positive

## References

[1] Siegel RL, Giaquinto AN, Jemal A (2024) Cancer statistics, 2024. CA Cancer J Clin 74:12–49 38230766 10.3322/caac.21820

[2] Nicholson AG, Tsao MS, Beasley MB et al (2015) The 2021 WHO classification of lung tumors: impact of advances since 2015. J Thorac Oncol 17:362–387 10.1016/j.jtho.2021.11.003 34808341

[3] Tsao MS, Nicholson AG, Maleszewski JJ et al (2022) Introduction to 2021 WHO classification of thoracic tumors. J Thorac Oncol 17:e1–e4 34930611 10.1016/j.jtho.2021.09.017

[4] Hansell DM, Bankier AA, MacMahon H et al (2008) Fleischner Society: glossary of terms for thoracic imaging. Radiology 246:697–722 18195376 10.1148/radiol.2462070712

[5] MacMahon H, Naidich DP, Goo JM et al (2017) Guidelines for management of incidental pulmonary nodules detected on CT images: from the Fleischner Society 2017. Radiology 284:228–243 28240562 10.1148/radiol.2017161659

[6] Lee SM, Park CM, Goo JM et al (2010) Transient part-solid nodules detected at screening thin-section CT for lung cancer: comparison with persistent part-solid nodules. Radiology 255:242–251 20173104 10.1148/radiol.09090547

[7] Qi LL, Wu BT, Tang W et al (2020) Long-term follow-up of persistent pulmonary pure ground-glass nodules with deep learning-assisted nodule segmentation. Eur Radiol 30:744–755 31485837 10.1007/s00330-019-06344-z

[8] Eguchi T, Kondo R, Kawakami S et al (2014) Computed tomography attenuation predicts the growth of pure ground-glass nodules. Lung Cancer 84:242–247 24681281 10.1016/j.lungcan.2014.03.009

[9] Fan L, Fang M, Li Z et al (2019) Radiomics signature: a biomarker for the preoperative discrimination of lung invasive adenocarcinoma manifesting as a ground-glass nodule. Eur Radiol 29:889–897 29967956 10.1007/s00330-018-5530-z

[10] Van Schil PE, Asamura H, Rusch VW et al (2012) Surgical implications of the new IASLC/ATS/ERS adenocarcinoma classification. Eur Respir J 39:478–486 21828029 10.1183/09031936.00027511

[11] Boland JM, Froemming AT, Wampfler JA et al (2016) Adenocarcinoma in situ, minimally invasive adenocarcinoma, and invasive pulmonary adenocarcinoma—analysis of interobserver agreement, survival, radiographic characteristics, and gross pathology in 296 nodules. Hum Pathol 51:41–50 27067781 10.1016/j.humpath.2015.12.010

[12] Hsu WC, Huang PC, Pan KT et al (2021) Predictors of invasive adenocarcinomas among pure ground-glass nodules less than 2 cm in diameter. Cancers (Basel) 13:3945 34439100 10.3390/cancers13163945 PMC8391557

[13] Xiong Z, Jiang Y, Che S et al (2021) Use of CT radiomics to differentiate minimally invasive adenocarcinomas and invasive adenocarcinomas presenting as pure ground-glass nodules larger than 10mm. Eur J Radiol 141:109772 34022476 10.1016/j.ejrad.2021.109772

[14] Sun Y, Li C, Jin L et al (2020) Radiomics for lung adenocarcinoma manifesting as pure ground-glass nodules: invasive prediction. Eur Radiol 30:3650–3659 32162003 10.1007/s00330-020-06776-y PMC7305264

[15] LeCun Y, Bengio Y, Hinton G (2015) Deep learning. Nature 521:436–444 26017442 10.1038/nature14539

[16] Murphy A, Skalski M, Gaillard F (2018) The utilisation of convolutional neural networks in detecting pulmonary nodules: a review. Br J Radiol 91:20180028 29869919 10.1259/bjr.20180028 PMC6350496

[17] He K, Zhang X, Ren S et al (2016) Deep residual learning for image recognition. In: Proceedings of the IEEE conference on computer vision and pattern recognition. IEEE, Las Vegas, pp 770–778

[18] Zhang S, Fan R, Liu Y et al (2023) Applications of transformer-based language models in bioinformatics: a survey. Bioinform Adv 3:vbad001 36845200 10.1093/bioadv/vbad001 PMC9950855

[19] Hu J, Zheng S, Wang B et al (2022) Super-resolution Swin Transformer and attention network for medical CT imaging. Biomed Res Int 2022:4431536 36531651 10.1155/2022/4431536 PMC9754833

[20] Bankier AA, MacMahon H, Colby T et al (2024) Fleischner Society: glossary of terms for thoracic imaging. Radiology 310:e232558 38411514 10.1148/radiol.232558 PMC10902601

[21] Yang J, Shi R, Wei D et al (2023) MedMNIST v2—a large-scale lightweight benchmark for 2D and 3D biomedical image classification. Sci Data 10:41 36658144 10.1038/s41597-022-01721-8 PMC9852451

[22] Jin L, Yang J, Kuang K et al (2020) Deep-learning-assisted detection and segmentation of rib fractures from CT scans: development and validation of FracNet. EBioMedicine 62:103106 33186809 10.1016/j.ebiom.2020.103106 PMC7670192

[23] Garfield DH, Cadranel JL, Wislez M et al (2006) The bronchioloalveolar carcinoma and peripheral adenocarcinoma spectrum of diseases. J Thorac Oncol 1:344–359 17409882

[24] Lim HJ, Ahn S, Lee KS et al (2013) Persistent pure ground-glass opacity lung nodules ≥ 10 mm in diameter at CT scan: histopathologic comparisons and prognostic implications. Chest 144:1291–1299 23722583 10.1378/chest.12-2987

[25] De Oliveira Duarte Achcar R, Nikiforova MN, Yousem SA (2009) Micropapillary lung adenocarcinoma: EGFR, K-ras, and BRAF mutational profile. Am J Clin Pathol 131:694–700 19369630 10.1309/AJCPBS85VJEOBPDO

[26] Bi WL, Hosny A, Schabath MB et al (2019) Artificial intelligence in cancer imaging: clinical challenges and applications. CA Cancer J Clin 69:127–157 30720861 10.3322/caac.21552 PMC6403009

[27] Li W, Cao P, Zhao D et al (2016) Pulmonary nodule classification with deep convolutional neural networks on computed tomography images. Comput Math Methods Med 2016:6215085 28070212 10.1155/2016/6215085 PMC5192289

[28] Shen D, Wu G, Suk HI (2017) Deep learning in medical image analysis. Annu Rev Biomed Eng 19:221–248 28301734 10.1146/annurev-bioeng-071516-044442 PMC5479722

[29] Liu Z, Lin Y, Cao Y et al (2021) Swin Transformer: hierarchical vision transformer using shifted windows. In: Proceedings of the IEEE/CVF international conference on computer vision. IEEE, Montreal, pp 10012–10022

[30] Qi L, Xue K, Li C et al (2019) Analysis of CT morphologic features and attenuation for differentiating among transient lesions, atypical adenomatous hyperplasia, adenocarcinoma in situ, minimally invasive and invasive adenocarcinoma presenting as pure ground-glass nodules. Sci Rep 9:14586 31601919 10.1038/s41598-019-50989-1 PMC6786988

[31] Zhao W, Xu Y, Yang Z et al (2019) Development and validation of a radiomics nomogram for identifying invasiveness of pulmonary adenocarcinomas appearing as subcentimeter ground-glass opacity nodules. Eur J Radiol 112:161–168 30777206 10.1016/j.ejrad.2019.01.021

